# No Placebo Effect beyond Regression to the Mean on the Six Minute Walk Test in Pulmonary Arterial Hypertension Trials

**DOI:** 10.3390/ijms24021069

**Published:** 2023-01-05

**Authors:** Dennis Anheyer, Till Johannes Bugaj, Rainer Lüdtke, Sebastian Appelbaum, Hubert Trübel, Thomas Ostermann

**Affiliations:** 1 Department for Psychology and Psychotherapy, Witten/Herdecke University, 58458 Witten, Germany; 2 Institute for General Practice and Interprofessional Care, University Hospital Tübingen, 72076 Tübingen, Germany; 3 Department of General Internal Medicine and Psychosomatics, University of Heidelberg, Medical Hospital, 69120 Heidelberg, Germany; 4 Stifterverband, 45128 Essen, Germany; 5 Department for Medicine, Witten/Herdecke University, 58458 Witten, Germany

**Keywords:** regression to the mean, placebo effect, 6-minute walking test, pulmonary arterial hypertension

## Abstract

In drug studies, patients are often included when the disease activity is high. This will make any treatment appear to lessen disease activity, although the improvement is biased by selection. This effect is known as regression towards the mean (RTM). We aimed at investigating drug trials in Pulmonary Arterial Hypertension (PAH) using the 6-minute walking distance test (6MWD) as a primary outcome for the phenomenon of RTM. An existing registry of 43 open label studies and 23 randomized controlled trials conducted between 1990 and 2009 was used as the data source. Data analysis was carried out for 18 randomized controlled trials (RCTs) and 24 open label studies out of this registry. Data were analyzed for verum and placebo arms of the RCTs separately, as well as for the open label arms. In the verum arms, the overall effect given as 33.2 m (95% CI: 25.7; 40.6]); 6MWD was slightly lower than the effect in the observational studies, with 44.6 m (95% CI: [25.4; 63.8]). After studying and interpreting the data, we found that regression towards the mean plays only a minor role in PAH studies. In particular, placebo effects in the RCTs were negligibly small, with a mean 6MWD of −2.5 m (95% CI: [−9.8; 4.7]) in the placebo arm. Therefore, our analysis indicates that results of non-randomized observational studies can be regarded as valid tools for gaining valid clinical effects in patients with PAH.

## 1. Introduction

Pulmonary arterial hypertension (PAH) is a rare disease in which patients suffer from progressive loss of their pulmonary vascular bed, leading to elevated pressure in the pulmonary circulation and subsequent right heart failure [1]. Usually, PAH patients are clinically classified according to the New York Heart Association (NYHA) categorization, in four stages depending on the degree of dyspnea in relation to the activity level (NYHA level I–IV). In the initial diagnosis, however, most patients present with unspecific symptoms, which can delay the final diagnosis [2]. Currently, there is still an average time from onset of symptoms to diagnosis of several years, given the unspecific symptoms of PAH, which impacts the mean survival time [3].

As a consequence, PAH patients in studies are more likely to show later disease stages of NYHA II or even NYHA III. Thus, it appears ethically difficult to justify a placebo group and this has fostered the conduction of uncontrolled observational studies, comparing only pre-/post-treatment effects [4].

In both controlled and uncontrolled drug trials, the distance the patient is able to walk in six minutes (6MWD) has generally been used as a functional primary outcome parameter [5]. However, the lack of controls and a possible selection bias might render the 6MWD-test susceptible to the statistical phenomenon of regression to the mean (RTM).

RTM, first described by Galton in 1886, is a statistical phenomenon of changes in repetitive measurements, simply because subjects with the highest or lowest baseline values were chosen [6,7]. In the case of uncontrolled clinical studies, this makes any treatment appear to lessen disease activity, although an improvement could be regarded as a “statistical consequence” not related to the treatment effect itself. Furthermore, some authors even argue that “improvements attributed to the placebo effect are actually instances of statistical regression” [8].

The aim of the current study was to simulate and analyze whether observational studies in PAH using the 6MWD-test show a “vulnerability” to RTM and whether a significant amount of placebo effect remained after correction for RTM. Therefore, an existing registry of 43 open label studies and 25 randomized controlled trials conducted between 1990 and 2010 was analyzed.

## 2. Results

In the study pool, the oldest observational study dates back to the year 1994, while the oldest including RCT was published in 1990. The vast majority of RCTs (*n* = 25; 80%) were published after 2003. Seventeen of the observational studies were either identified as mono- or multicenter studies (39.5% each). The remaining nine studies (21%) were not clearly labeled as mono- or multicenter. Among the 25 RCTs, 18 (72%) were multicenter studies, and only five were clearly labeled as monocentric (20%). Two studies were not clearly labeled as either mono- or multicentric. In accordance with the late diagnosis of PAH, patients of all four NYHA classes were represented in the selected studies, but overall the majority was to be found in either functional class II or III.

From the 43 observational studies, 50 different “study arms” (each with individual 6MWD data) could be extracted, while the 25 RCTs consisted of 38 different treatment arms, mainly due to different dosing in parallel groups, plus a corresponding placebo (or control) arm.

The average mean age from the verum arms of the RCTs was 49.1 ± 7.2 years which corresponded well with the average mean age of the placebo groups (49.1 ± 9.15 years). The average mean age from all cohort study arms was younger, with a mean of 41.3 ± 14.1 years. In the 50 arms of the observational study group, Bosentan (*n* = 14; 28%) and Sildenafil (*n* = 12; 24%) were commonly used as therapy, but more uncommon therapeutic options such as Treprostinil (*n* = 3; 6%) or even the infusion of endothelial progenitor cells (*n* = 1; 2%) were also investigated.

In the 38 arms of the selected RCTs, Bosentan (*n* = 8; 21.1%) and Sildenafil (*n* = 9; 23.7%) were often used as study medication, but Ambrisentan (*n* = 8; 21.1%) was also used frequently, which played no role in the selected observational studies.

**Figure 1:**
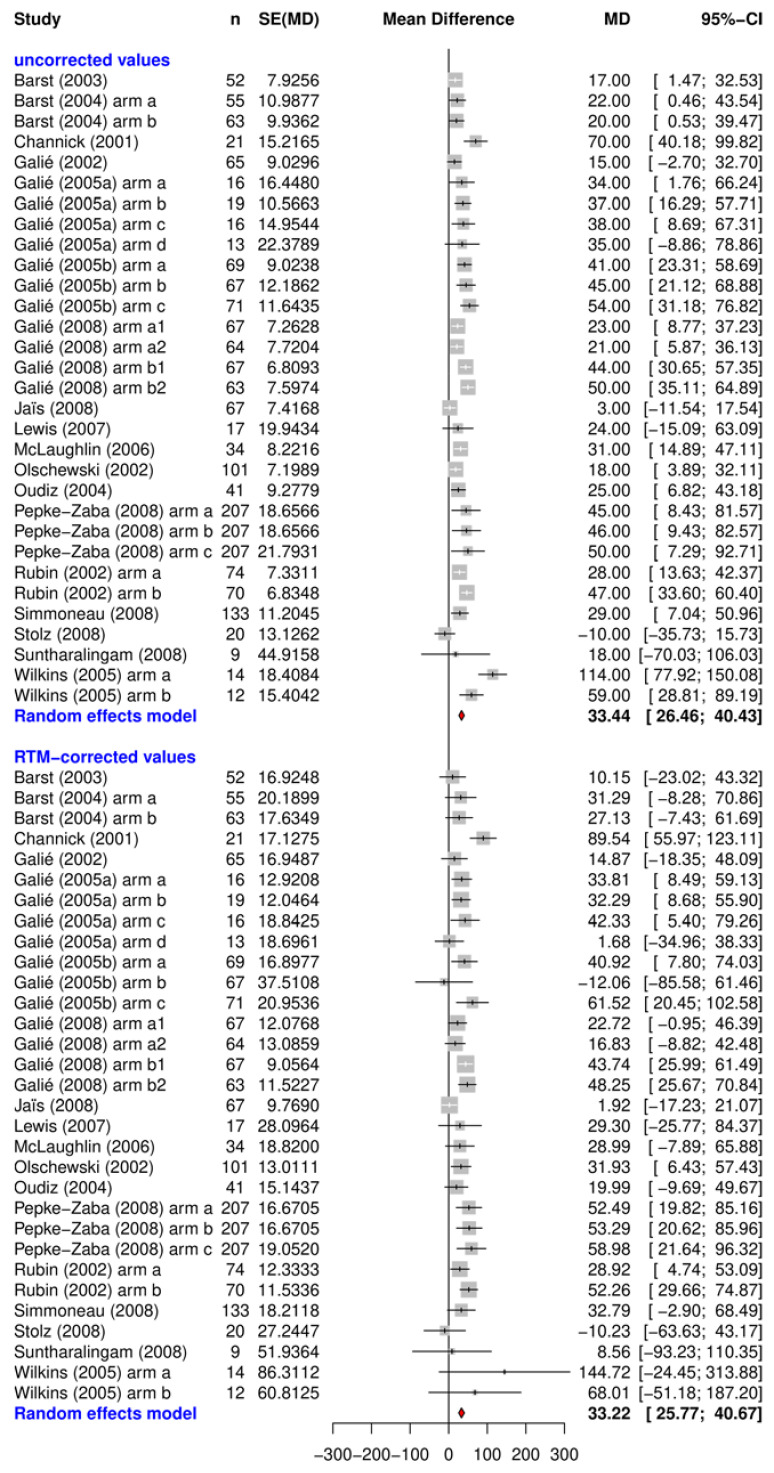
Uncorrected and RTM-corrected treatment effects measured with the 6MWD after 12 weeks from the verum arms. Abbreviations: n = number of participants in study (arms); seTE = standard error of treatment effect; MD = mean difference; 95% CI = 95% confidence interval. Note: the difference in numbers between the placebo and the verum group in Figure 1 and Figure 2 can be explained by the fact that many studies compared several treatment groups to only one single placebo group.

**Figure 2:**
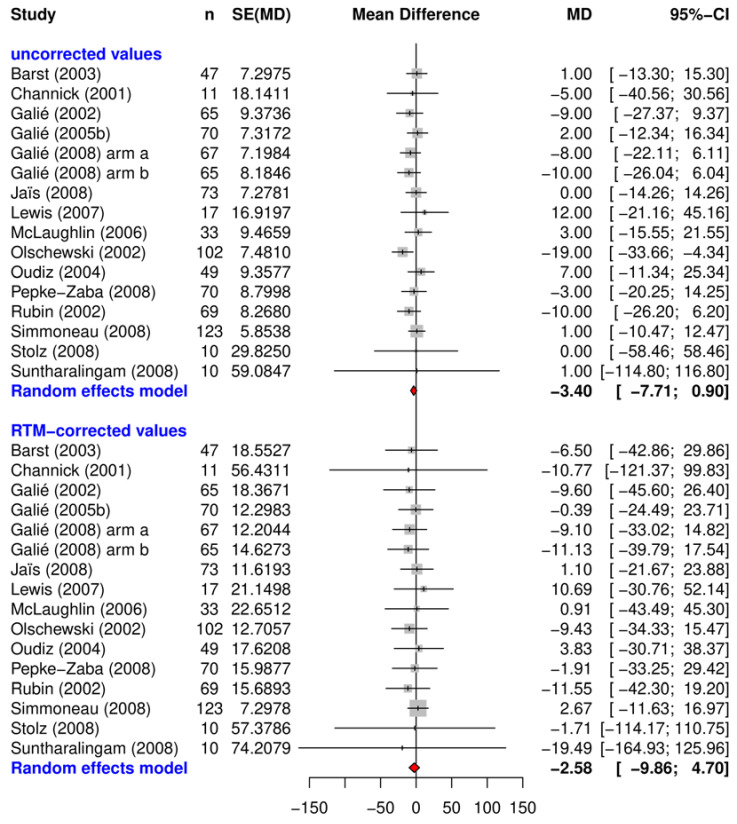
Uncorrected and RTM-corrected treatment effects measured with the 6MWD after 12 weeks from the placebo arms. Abbreviations: n = number of participants in study (arms); seTE = standard error of treatment effect; MD = mean difference; 95% CI = 95% confidence interval. Note: the difference in numbers between the placebo and the verum group in Figure 1 and Figure 2 can be explained by the fact that many studies compared several treatment groups to only one single placebo group.

**Figure 3:**
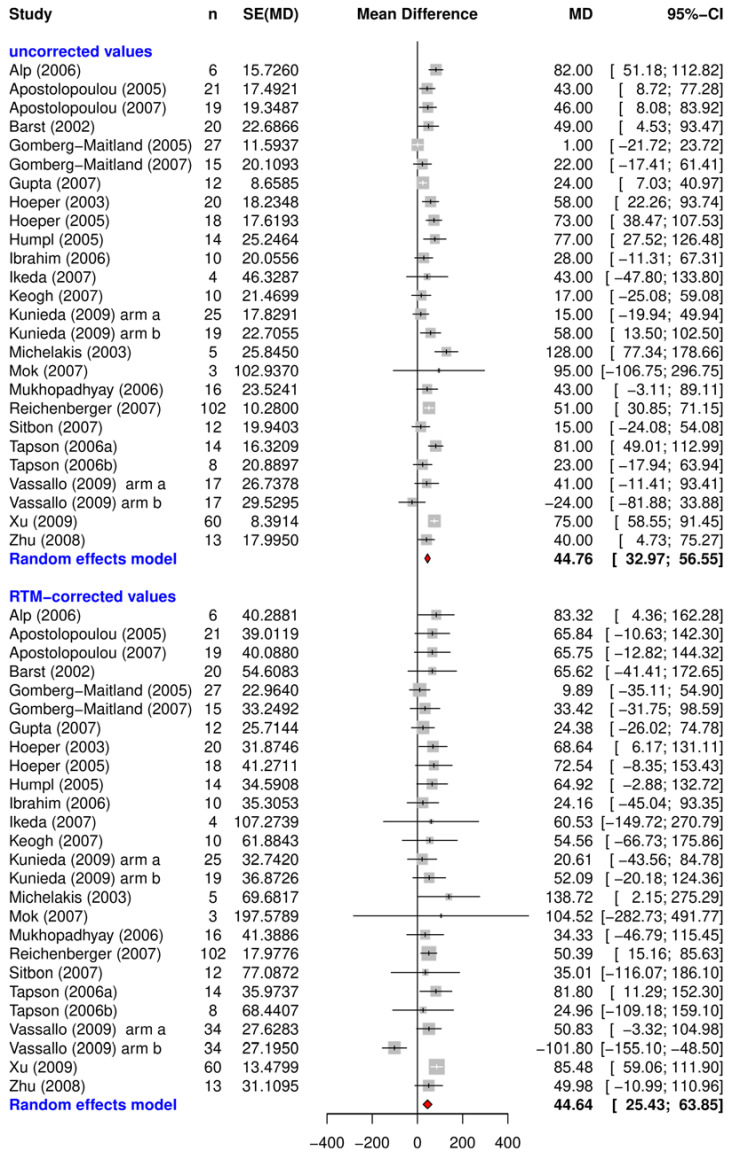
Uncorrected and RTM-corrected treatment effects measured with the 6MWD after 12 weeks from open label studies. Abbreviations: n = number of participants in study (arms); seTE = standard error of treatment effect; MD = mean difference; 95% CI = 95% confidence interval.

The uncorrected and RTM-corrected treatment effects after 12 weeks with their related confidence-intervals are shown in Figure 1, Figure 2 and Figure 3. They each represent data from the placebo arms of the RCTs (Figure 2), the observational studies (Figure 3), and the verum arms of the RCTs (Figure 1).

The results of the open label trials are mainly positive, with only one obvious outlier [9]. Besides this outlier, RTM-adjusted treatment effects vary from 9.9 m to 138.7 m with an overall mean of 44.6 m (95% CI: [25.4; 63.8]). Data from the verum groups included no negative outliers. In the verum arms the overall effect, given as 33.2 m (95% CI: [25.7 40.6]), was slightly lower than the effect in the observational studies.

Analysis of the placebo-study arms of the RCTs obviously differs from the aforementioned graphs: the effects of the 16 placebo arms generally assemble very close to the “zero-effect” line, meaning that the average patient hardly improved in walking performance without treatment. In several studies, a deterioration in the walking distance 12 weeks after the baseline data was even observed, which is also indicated by the overall effect of −2.5 m (95% CI: [−9.8; 4.7]).

## 3. Discussion

To our knowledge, this analysis is the first to compare RTM-adjusted 6MWD results of PAH patients included in RCTs with those included in observational studies. We found similar effects in observational studies and treatment arms of RCTs. Furthermore, the analysis of the placebo group in RCTs revealed a neglectable placebo effect.

The 6MWD is an often-used primary end point in heart failure or PAH studies and remains a label relevant endpoint for pharmacological studies in PAH [10]. The same applies for the European Agency for the Evaluation of Medicinal Products (EMEA) and their regulations on PAH drugs [4]. The 6MWD is easy to perform in patients with PAH and is applicable to most age groups. Its reproducibility in individual patients makes the 6MWD a standard biomarker for routine assessment of functional capacity [11]. Nevertheless, it is often suggested that the 6MWD is not sufficiently “robust” to describe the effect of therapy, as its value is, for instance, hampered in patients whose exercise performance is limited by comorbidities [12].

Considering this background, our results support the view by Halpern et al. that placebo-controlled trials in treatment-naïve patients with PAH are of limited use due to the progressive nature of the underlying disease [13]. Since we found only a minimal placebo effect size in PAH studies, i.e., patients in the placebo arm of RCT will hardly have any benefit from being randomized into the placebo group, several authors have argued that it appears difficult to justify a placebo group without treatment [14]. This clearly contrasts with studies with a focus on less severe conditions, e.g., arterial hypertension or mild asthma, where temporarily withholding an active treatment from a placebo group appears to be less harmful.

According to our analysis, the 6MWD appears to be unaffected by RTM since the RTM-adjusted analysis still shows a treatment effect of similar size when compared to RCTs.

From the methodological point of view, various methods for detecting RTM have been developed, both for cases of normal distributed data as well as for non-parametric testing [7]. Most of these methods deal with common situations of truncated sampling, i.e., only those members which have a first measurement beyond (or below) a predefined cut-off point are sampled.

In our approach, we are able to detect RTM simply by adjusting the therapeutic effect to the mean of an unselected population. This method, originally proposed by Mee and Chua, can also be applied as a modified t-test and offers a promising potential for detecting, distinguishing and quantifying the amount of RTM effects [15].

### Limitations

Limitations that are inherent to the design of our study are the publication- and retrieval-bias. Furthermore, our RTM-analysis was entirely based on 6MWD-values published by Humbert et al., which could have a center-related bias [16].

It has been shown before that there are geographic variations in 6MWD and to some degree caution must be taken when using predictive algorithms, as the included study populations might differ from each other as well as from the population which was used for the creation of the algorithm in terms of baseline walking distances [17].

There are also some limitations due to the different structures of the various original studies that we included into our analysis: some of the data presented above are based on single study arms, rather than total studies, as some studies included different etiologies in their individual arms, whereas in other cases no different individual arms were present.

Some baseline characteristics were hard to assess (e.g., NYHA-classes, age, etc.), as some of the included study arms referred to the same population as the authors did not point out an individual baseline dataset for each and every subgroup, meaning that the calculated average values are only as reliable as the original data supplied by the various authors. Some authors even supplied the same baseline data for both the verum and the placebo group in their RCT. Even worse, some baseline characteristics such as age were not even universally listed in every single study. Additionally, the forest plots in our analysis only display the effects after 12 weeks, meaning that not every single study/study arm included in our analysis is also to be found in one of these plots.

Finally, although this study was solely carried out to analyze the RTM-effect, our data pool does not include actual study data and, thus, to underpin our findings, an analysis including also actual studies should be rolled out. Additionally, it should be mentioned that this research was not aimed as a systematic review. Therefore, it does not follow the PRISMA guidelines and selection bias cannot be ruled out completely.

## 4. Materials and Methods

An existing registry of 43 open label studies and 25 randomized controlled trials conducted between 1990 and 2010 was used as the data source. This study pool was initially created by systematically searching the PubMed database for studies using search terms around “pulmonary hypertension” AND “6 MWD”. Studies with both adult and pediatric patients with pulmonary hypertension of all etiologies (PAH, PH secondary to other medical conditions) were included. Studies assessing the walking distance only once (e.g., at the beginning of the study) were excluded. Finally, a total of 18 randomized controlled trials and 24 open label studies were included in the quantitative analysis [9,18,19,20,21,22,23,24,25,26,27,28,29,30,31,32,33,34,35,36,37,38,39,40,41,42,43,44,45,46,47,48,49,50,51,52,53,54,55,56,57,58].

If trials included more than one interventional group (and thus more than one set of pre- and post-interventional 6-MWD-data), we included data from all study arms. The following data were extracted independently by two of the authors: year of publication, study type (Uncontrolled vs. RCT), number of study centers. For each arm, the number of patients, patients’ age (Mean ± SD), treatment, and 6MWD test results (Mean ± SD; SE) were extracted.

Data were separately analyzed by type of study and type of intervention (verum, placebo). For each study/intervention we calculated the RTM corrected effect size *τ* according to the formula

(1) $$\tau ={\bar{x}}_{2}-\rho \left({\bar{x}}_{1}-\mu \right)-\mu$$

given in [59], originating from the modified paired sample t-test developed by Mee and Chua where ${\bar{x}}_{1}$ and ${\bar{x}}_{2}$ describe the 6MWT means before and after the intervention, *ρ* denotes the correlation coefficient between pre- and post-values, and *µ* is the mean of the 6MWT in the (unselected) population of all PAH patients [60]. In cases of unknown *ρ*, its value was set to 0.7. The value for *µ* was obtained for each study as a weighted mean of 6MWT normal values according to patients’ gender and NYHA classification data provided in [16].

The overall RTM adjusted treatment effects were estimated by random effect meta-analysis using the R-package Meta [61]. Results of the meta-analysis are presented as forest plots for both the RTM-adjusted and unadjusted effects and displayed for each study in alphabetical order. The individual measure of effect is represented as mean difference (MD) with a corresponding 95% confidence interval. In this case, the MD represents the difference between the baseline measurement and 12 week follow up measurement for each study and study arm.

## 5. Conclusions

Our results suggest that, in general, the 6MWD is a valuable marker for PAH studies and is not remarkably affected by RTM according to our meta-analysis. Our data analysis supports the notion that treatment-naïve PAH patients do not profit from RCTs when they are included in the placebo group. Since most patients that presently enter PAH studies are already on multiple treatments, adaptive trial designs, i.e., with interim analysis, as suggested in the literature, might be suited to minimize the time needed to show a positive effect of a new regime added to the standard of care [15].

## Data Availability

Not applicable.
